# Evaluating informed consent during trial recruitment consultations: development and preliminary evaluation of a new method

**DOI:** 10.1186/1745-6215-16-S2-P118

**Published:** 2015-11-16

**Authors:** Julia Wade, Daisy Townsend, Daisy Gaunt, Kerry Avery, Grace Young, Jenny Donovan

**Affiliations:** 1 University of Bristol, Bristol, UK

## Background

Informed consent (IC) is legally and ethically required for trial participation. Evidence suggests participants do not always fully understand information before consenting. Systematic reviews highlight benefits of discussion to enhance understanding and call for standardisation when evaluating IC. We describe the development and preliminary evaluation of a novel measure for application to audio-recordings of recruitment consultations that aims to evaluate recruiter information provision and patient understanding.

## Methods

Essential components of informed consent identified in the literature informed item development. A measure comprising 20 items assessing recruiter information provision and patient understanding and 14 items assessing recruiter/patient interaction was applied to six audio-recorded recruitment consultations from three trials by two raters. Validity and reliability were evaluated as follows: feasibility (time to complete), content validity (response rates) construct validity (comparing scores on the measure with assessment on three key parameters), test-retest reliability (with 14 day interval) and inter-rater reliability. Descriptive analyses were undertaken.

## Results

Items included discussion of diagnosis, management options, equipoise, trial purpose, randomisation, treatments and procedures. The measure took a mean of 97 (range 38-169) minutes to complete. Comparison of response rates, test-retest and inter-rater reliability agreement identified items for removal/modification. The revised measure comprised evaluation of recruiter information provision and patient understanding (15 items) and judgements about patient understanding at the start and close of the discussion (6 items).

## Conclusions

The revised measure represents a novel, feasible method of evaluating recruiter information provision and patient understanding during trial recruitment discussions. Further evaluation of validity and reliability is ongoing.

